# Complementary Activities of Bacteriophages and Antimicrobial Peptide Dendrimers Against Prosthetic Joint Infection Pathogens

**DOI:** 10.3390/bioengineering13080870

**Published:** 2026-07-28

**Authors:** Shawna McCallin, Caroline Lanz, Sandra Jaccoud, Alexis E. Laurent, Lee Ann Applegate, Philippe Abdel-Sayed

**Affiliations:** 1 Regenerative Therapy Unit, Lausanne University Hospital (CHUV), 1011 Lausanne, Switzerland; shawna.mccallin@balgrist.ch (S.M.); lanz.caroline.ch@gmail.com (C.L.); sandra.jaccoud@epfl.ch (S.J.); lee.laurent-applegate@unil.ch (L.A.A.); 2 Department of Neuro-Urology, Balgrist University Hospital, University of Zurich, 8008 Zurich, Switzerland; 3 Spinal Cord Injury Center, Balgrist University Hospital, University of Zurich, 8008 Zurich, Switzerland; 4 Manufacturing Department, Tec-Pharma SA, 1038 Bercher, Switzerland; alexis.laurent@pharmabercher.ch; 5 Discovering Learning Labs, Ecole Polytechnique Fédérale de Lausanne (EPFL), 1015 Lausanne, Switzerland

**Keywords:** prosthetic joint infection, bacteriophages, antimicrobial peptide dendrimers, orthopedic biomaterials, biofilm-associated infections, antimicrobial resistance, implant coatings

## Abstract

Prosthetic joint infections (PJIs) remain a major challenge in orthopedic surgery due to the increasing prevalence of antimicrobial-resistant pathogens and their ability to form biofilms on implant surfaces. Local delivery of non-traditional antimicrobials through implant-associated biomaterials represents a promising strategy for preventing bacterial colonization while minimizing systemic antibiotic exposure. This study evaluated the antimicrobial activity and cytocompatibility of two bacteriophages (Phage K and Phage F1) and two antimicrobial peptide dendrimers (AMPDs; TNS18 and G3KL) against clinical isolates of *Staphylococcus aureus* and *Staphylococcus epidermidis* recovered from PJIs. Antimicrobial efficacy was assessed using solid and liquid culture assays, while cytocompatibility was evaluated using human osteoblast progenitor cells. Biofilm formation was also investigated under various in vitro conditions. Phages K and F1 demonstrated strong antibacterial activity against *S. aureus* isolates, whereas TNS18 showed pronounced inhibitory effects against *S. epidermidis*. All agents exhibited appropriate osteoblast compatibility, except Phage F1, at the highest multiplicity of infection tested. Biofilm formation was observed under several culture conditions, although substantial variability in biofilm stability limited the quantitative assessment of antimicrobial activity. These findings demonstrate complementary antimicrobial activity profiles between bacteriophages and AMPDs and support their further investigation as candidates for implant-associated antimicrobial delivery systems and orthopedic implant coatings.

## 1. Introduction

Prosthetic joint infections (PJIs) remain among the most frequent complications of primary joint arthroplasties and one of the predominant reasons for early surgical revision. Thus, they constitute highly challenging complications in orthopedic surgery, often resulting in prolonged hospitalization, repeated surgical interventions, and implant failure [1]. These complications significantly affect patient outcomes and impose a substantial economic burden on healthcare systems [2].

The clinical management of PJIs is further complicated by their increasing frequency as well as the emergence of antibiotic-resistant pathogens and the formation of biofilms on implant surfaces, both of which limit the effectiveness of conventional DAIR (debridement, antibiotics, and implant retention) approaches and other surgical therapies [3,4,5]. *Staphylococcus aureus* and *Staphylococcus epidermidis* are responsible for most PJIs due to their ability to adhere to metallic surfaces and establish persistent biofilms [6,7,8]. Once formed, biofilms markedly reduce bacterial susceptibility to antibiotics by creating a physical barrier and promoting slow-growing phenotypes, often necessitating aggressive surgical management or implant removal [5,9]. Concurrently, the global rise of multidrug-resistant (MDR) bacteria has intensified concerns about limited treatment options. The World Health Organization (WHO) identifies antimicrobial resistance (AMR) as one of the top ten global public health threats, with deaths attributable to drug-resistant infections projected to increase dramatically in the coming years and has urged the development of novel and non-traditional antimicrobial (NTA) strategies [10,11].

Recent advances in biomaterials and regenerative medicine have enabled the development of multifunctional orthopedic implants designed not only to restore mechanical function, but also to actively modulate the biological environment surrounding the implant [12,13]. Antimicrobial coatings, hydrogels, and controlled-release systems represent promising approaches to reduce infection risks while simultaneously supporting tissue integration and bone regeneration. The success of such technologies, however, depends on the availability of antimicrobial agents that combine potent antibacterial activity with excellent cytocompatibility toward host cells involved in osseointegration and tissue repair or toward exogenous therapeutic cellular payloads.

Bacteriophages have re-emerged as promising candidates in the search for alternatives to classical antibiotics, particularly for osteo-articular infections like PJIs [14,15]. These viruses specifically target and lyse bacteria, including MDR strains, while sparing mammalian cells as well as the commensal microbiota [16,17]. Several studies have shown that bacteriophages can penetrate and disrupt biofilms, making them particularly attractive for implant-associated infections [18,19]. Phage K, a well-characterized lytic myovirus phage active against *S. aureus*, has demonstrated broad-host-range activity and rapid bactericidal effects [20,21]. Phage F1, which is a similarly related myovirus, was isolated from a Georgian phage cocktail, Fersisi, demonstrating its therapeutic use and potential. A previous metagenomic analysis of phage preparations from Eastern Europe demonstrated the high similarity of staphylococcal phages included in therapeutic preparations [22,23,24].

Antimicrobial peptide dendrimers (AMPDs) represent another promising class of emerging antimicrobial agents relevant to implant-associated infections. Dendrimers are synthetic molecules characterized by a tree-like branched architecture that can be precisely engineered to modulate biological properties [25]. Through a combination of topology and sequence design, several AMPDs active against MDR isolates of *Pseudomonas aeruginosa* and *Acinetobacter baumannii* have been developed [26]. Among these, G3KL and G3RL have been preclinically studied in the context of biological bandages for burn wound treatment, where they successfully prevented *P. aeruginosa* infections without impairing therapeutic cell viability, and additionally demonstrated angiogenic effects that support tissue repair [27]. More recently, Siriwardena and colleagues developed a smaller lipidated AMPD, TNS18, structurally related to G3KL but exhibiting broader antimicrobial activity against MDR *P. aeruginosa*, *A. baumannii*, and methicillin-resistant *S. aureus* (MRSA) [28]. Its mechanism of action involves rapid bacterial membrane disruption, leading to cell death. Upon contact with the bacterial membrane, the dendrimer unfolds, exposing its lipid chain and hydrophobic residues, thereby promoting membrane destabilization and permeabilization. These findings highlight the strong potential of AMPDs for preventing implant colonization. Nevertheless, their activity against clinically derived PJI isolates and their compatibility with human therapeutic progenitor osteoblasts remain insufficiently explored.

Despite increasing interest in both bacteriophages and AMPDs, few studies have directly compared these two antimicrobial classes or examined their potential combination for preventing PJIs. Furthermore, only limited experimental work has evaluated their performance under conditions mimicking implanted orthopedic biomaterials.

The present study therefore evaluated the antimicrobial activity and cytocompatibility of two bacteriophages (Phage K and Phage F1) and two AMPDs (TNS18 and G3KL) against clinical isolates of *S. aureus* and *S. epidermidis* obtained from clinical cases of PJIs. In addition, preliminary biofilm models were investigated to better reproduce conditions encountered on orthopedic implants. By identifying antimicrobial agents that combine efficacy against PJI-associated pathogens with compatibility toward osteogenic cells, this work contributes to the development of next-generation bioengineered implant coatings and local antimicrobial delivery strategies for infection prevention.

## 2. Materials and Methods

### 2.1. Bacterial Isolates and Antimicrobial Agents

Three clinical isolates each of *S. aureus* and *S. epidermidis* were obtained from prosthetic joint fluid samples collected at the Lausanne University Hospital (CHUV, Lausanne, Switzerland). Two lytic bacteriophages, Phage K (kindly provided by Dr. Aidan Coffey, University College Cork, Ireland) and Phage F1 (isolated from a commercial Georgian phage cocktail), were propagated using *S. aureus* 8325-4 as the host strain. After amplification, phage lysates were sterile-filtered (0.22–0.44 mm). Phage stock concentrations were maintained at 1–5 × 10^9^ plaque-forming units (PFU)/mL.

The AMPDs G3KL and TNS18 were provided by Prof. Jean-Louis Reymond (Department of Chemistry and Biochemistry, University of Bern, Switzerland). Dendrimer synthesis was performed by solid-phase peptide synthesis as previously described [26], followed by purification using reverse-phase high-performance liquid chromatography (RP-HPLC). Lyophilized trifluoroacetate salts were dissolved in phosphate-buffered saline (PBS) to obtain stock solutions at a concentration of 20 mg/mL.

### 2.2. Cytocompatibility Assessment

Vials of cryopreserved FE002-Bone primary progenitor osteoblasts (1 × 10^6^ cells/mL frozen in DMEM, FBS, and DMSO at a ratio of 5:4:1) were obtained from the DAL biobank (Biobank DAL, Biobank of the Musculoskeletal Medicine Department of the Lausanne University Hospital, CHUV, [BB_029_DAL]) at low passages. Cells were used between passages 2–5 for experimentation.

The FE002-Bone primary human osteoblasts were thawed and seeded in 48-well plates at a density of 3000 cells/cm^2^ in DMEM with 10% FBS and 5% L-glutamine. For dendrimer testing, stock solutions were prepared at 200 μg/mL and subsequently diluted to final concentrations of 100, 50, and 25 μg/mL. Phages K and F1 were evaluated at multiplicities of infection (MOIs) of 1 and 0.1; MOI represents the ratio of phage particles to bacterial cells, where an MOI of 1 reflects equal numbers of phages and bacteria, and an MOI of 0.1 indicates that bacterial cells outnumber phages tenfold. The MOIs used for testing were selected based on therapeutic plausibility at expected sites of infection. Treatments were added 24 h after cell seeding (*n* = 6 wells per condition).

Cell viability was assessed on days 0, 1, 3, 7, 10, and 14 using the CellTiter 96^®^ Aqueous One Solution Cell Proliferation Assay (Promega, Madison, WI, USA) according to the manufacturer’s instructions. Briefly, a reagent solution was prepared by diluting 10 μL of CellTiter 96^®^ reagent in 100 μL of culture medium. Prior to the addition of the reagent solution, culture medium was aspirated, and the cells were washed once with 100 μL of phosphate-buffered saline (PBS; GIBCO PBS, pH 7.4, Life Technologies, Carlsbad, CA, USA). For negative controls, culture medium was removed and replaced with 100 μL of methanol 30 min before measurement. Subsequently, 100 μL of the reagent solution was added, and the plates were incubated for 90 min at 37 °C. Finally, 90 μL of supernatant was transferred to a 96-well plate and sample absorbance was measured at 490 nm using a Wallac Victor^2^ 1420 multilabel counter (PerkinElmer, Waltham, MA, USA).

### 2.3. Evaluation of Antimicrobial Activity

#### 2.3.1. Drop Test Assay

Antimicrobial activity was initially evaluated using soft-agar overlay assays. Briefly, 200 μL of an overnight (ON) bacterial culture grown in Brain Heart Infusion (BHI) medium was mixed with 4 mL of BHI soft agar maintained at 55 °C and poured onto BHI agar plates. Following solidification, 3.5 μL of 10-fold serial dilutions of stock bacteriophages prepared in 0.9% NaCl or AMPD solutions prepared in PBS were spotted onto the bacterial lawn. Plates were incubated ON at 37 °C, and plaque formation or growth inhibition zones were recorded the following day.

#### 2.3.2. Turbidity Reduction Assay (TRA)

Bacterial growth inhibition was assessed in flat bottom, microbiology culture-treated 96-well plates. For each condition, 12.5 μL of an ON bacterial culture was added to wells containing either bacteriophages or AMPDs. Phages were applied at MOIs of 1, 0.1, or 0.01. AMPDs (G3KL or TNS18) were tested in Brain Heart Infusion (BHI) medium at final concentrations of 100 μg/mL (G3KL and TNS18), 50 μg/mL (G3KL and TNS18), or 25 μg/mL (TNS18 only). The final volume in each well was 225 μL, representing a starting bacterial concentration of approximately 1–5 × 10^7^ CFU/mL. PBS served as the negative control. Plates were incubated in a microplate reader preheated to 37 °C, and bacterial growth was monitored by measuring optical density (OD) at 600 nm every 10 min for 20 h.

### 2.4. Biofilm Formation

Biofilm formation was evaluated under multiple culture conditions to establish an in vitro model using clinical *S. aureus* and *S. epidermidis* isolates. ON cultures were diluted and seeded into tissue-culture-treated 96-well polystyrene plates at final inoculum densities of 2 × 10^3^ or 2 × 10^4^ CFU per well in a total volume of 200 μL. Plates were incubated statically at 37 °C for either 24 or 48 h. For experiments exceeding 24 h, an additional 50 μL of fresh medium was added after the first 24 h incubation period.

The following culture media were evaluated: BHI (0.2% glucose, 0.5% NaCl), glucose-supplemented BHI (1% glucose, 0.5% NaCl), glucose-supplemented Tryptic Soy Broth (TSB; 1% glucose, 0.5% NaCl), BHI and TSB supplemented with 1% or 2% NaCl, and Dulbecco’s modified Eagle medium (DMEM) containing 10% FBS, 5% glutamine, 0.45% glucose, and 0.64% NaCl.

At the end of each experiment, culture medium was removed, and biofilms were fixed by drying at 60 °C for 1 h. Biofilms were stained with 75 μL of 0.1% crystal violet for 20 min at ambient temperature and subsequently washed three times with 100 μL distilled water.

To evaluate phage activity against established biofilms, samples grown for 24 h were exposed to 100 μL of Phage K or Phage F1 diluted in 0.9% NaCl at an MOI of 1 for 48 h. In a second experimental setup, biofilms were grown for 48 h before exposure to phages at MOIs of 1 or 0.1 for 5 h. Following treatment, biofilms were fixed and stained as described above. Due to the limited availability of AMPDs, dendrimer activity was not assessed in the biofilm experiments.

### 2.5. Statistical Analysis

When appropriate, data were expressed as the means ± SD. One-way ANOVA analysis was performed with Dunnet’s post hoc test. A value of *p* < 0.05 was considered statistically significant. Experiments were performed with *n* = 3–6 replicates.

## 3. Results

### 3.1. Cytocompatibility of Antimicrobial Candidates for Implant-Associated Delivery Systems

**Figure 1:**
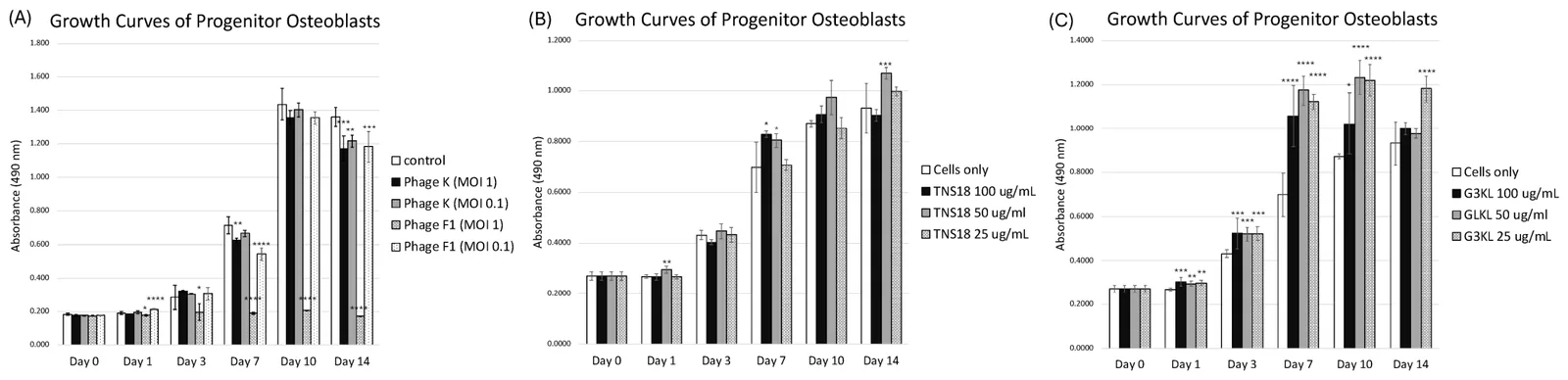
Effect of antimicrobial strategies on cell viability. (**A**) Growth curves of FE002-Bone progenitor osteoblasts cultured in the presence of bacteriophages K and F1 at MOIs of 1 and 0.1. (**B**) Growth curves of progenitor osteoblasts cultured in the presence of the AMPD TNS18 at concentrations of 100, 50, and 25 μg/mL, respectively. (**C**) Growth curves of progenitor osteoblasts cultured in the presence of the AMPD G3KL at concentrations of 100, 50, and 25 μg/mL, respectively. * *p* < 0.05; ** *p* < 0.01; *** *p* < 0.001; **** *p* ≤ 0.0001.

Cytotoxicity testing demonstrated appropriate biocompatibility for both AMPDs and bacteriophages, with one notable exception. Phage F1 at an MOI of 1 reduced osteoblast proliferation over the 14-day culture period, whereas Phage K showed no adverse effect on cell growth (Figure 1A). Both G3KL and TNS18 were well-tolerated by FE002-Bone progenitor osteoblasts (Figure 1B,C). Notably, G3KL promoted osteoblast proliferation from day 3 onward, suggesting a potential beneficial effect on cellular growth and tissue regeneration.

The favorable cytocompatibility profile observed for Phage K, TNS18, and G3KL supports their potential incorporation into biomaterial-based delivery platforms and antimicrobial implant coatings. In particular, the ability of G3KL to enhance osteoblast proliferation suggests additional value for applications aimed at simultaneously preventing infection and promoting peri-implant bone regeneration. Residual bacterial byproducts of phage lysis present in the applied lysate may have been the cause of the decreased cell viability following exposure to Phage F1, which could be remediated using phage purification techniques, such as tangential flow filtration, cesium-chloride density ultracentrifugation, or anion-exchange chromatography [29].

### 3.2. Identification of Antimicrobial Candidates for Implant-Associated Infection Prevention

**Figure 2:**
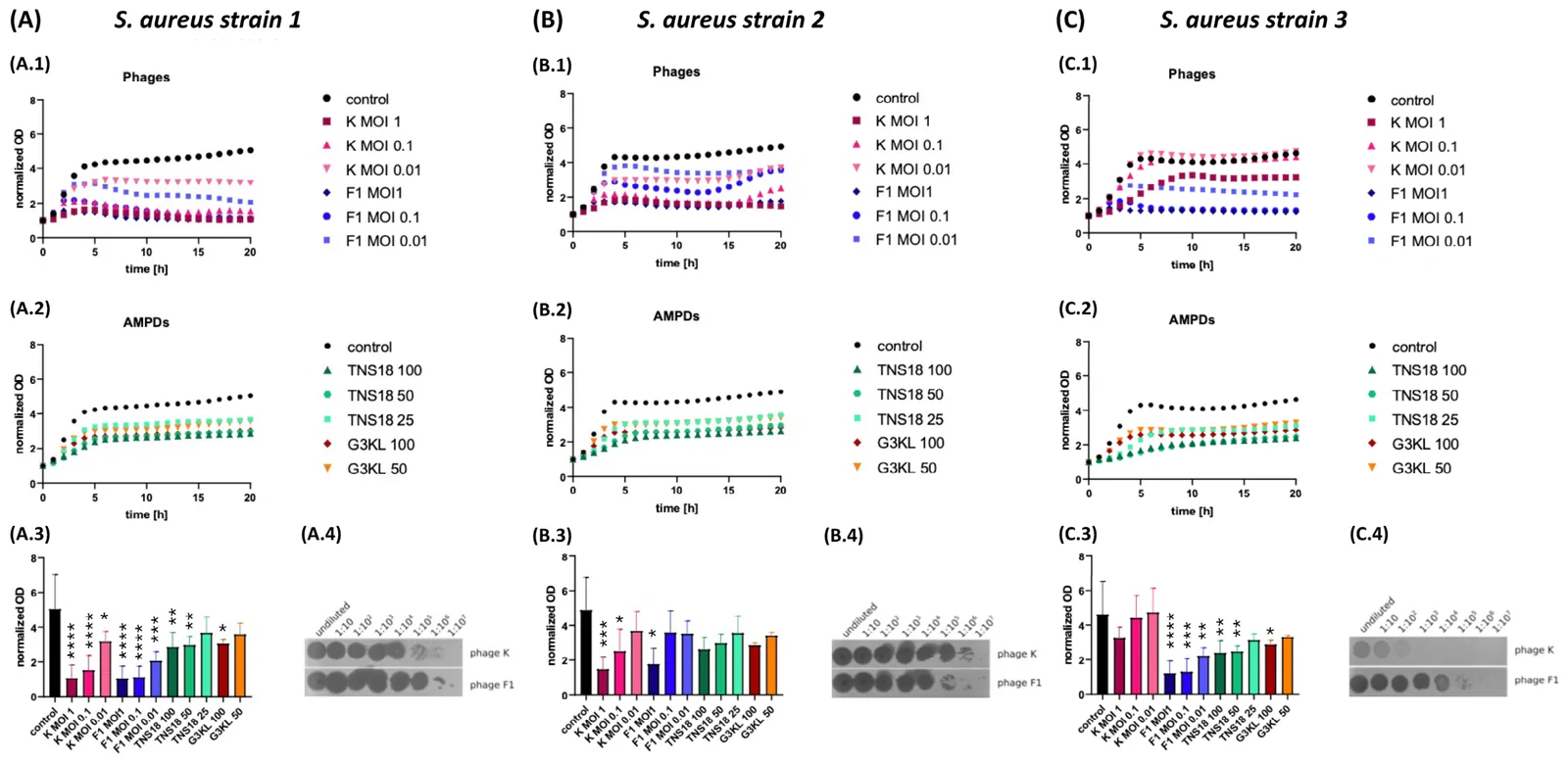
Susceptibility of *S. aureus* clinical isolates to bacteriophages and AMPDs. (**A.1**–**C.1**) Growth kinetics of *S. aureus* isolates incubated with Phage K or Phage F1 at MOIs of 1, 0.1, and 0.01, or without phage (control), over 20 h. OD_600_ was normalized to the initial value at t = 0 h. Data represent the mean of three biological replicates. (**A.2**–**C.2**) Growth kinetics of *S. aureus* isolates incubated with TNS18 (100, 50, and 25 μg/mL) or G3KL (100 and 50 μg/mL), or without AMPD (control), over 20 h. OD_600_ values were normalized to the initial OD_600_ value. Data represent the mean of three biological replicates. (**A.3**–**C.3**) Quantification of bacterial growth at 20 h for the conditions shown in panels (**A.1**–**C.1**). * *p* < 0.05; ** *p* < 0.01; *** *p* < 0.001; **** *p* ≤ 0.0001. (**A.4**–**C.4**) Drop test assay results showing the susceptibility of *S. aureus* isolates to serial dilutions of Phage K and Phage F1, starting from a concentration of 10^9^ PFU/mL. Drop test results for TNS18 (100, 50, and 25 μg/mL) and G3KL (100 and 50 μg/mL) are not shown due to bacterial overgrowth, which prevented reliable visualization of inhibition zones.

Clinical isolates of *S. aureus* (*n* = 3) and *S. epidermidis* (*n* = 3) obtained from clinical cases of PJIs were evaluated to identify antimicrobial agents suitable for future integration into implant-associated delivery systems. Both bacteriophages demonstrated pronounced activity against *S. aureus* isolates. In both liquid culture and agar-based assays, phage-mediated growth inhibition was dose-dependent, with the strongest antibacterial effect observed at an MOI of 1 (Figure 2). While *S. aureus* strains 1 and 2 were efficiently lysed by both phages, strain 3 exhibited differential susceptibility. Phage F1 produced clear lysis zones and reduced normalized bacterial growth by approximately 3.8-fold after 20 h, whereas Phage K induced only partial growth inhibition, resulting in a turbid lysis phenotype and approximately 30% reduction in bacterial growth at an MOI of 1 (Figure 2C). These findings indicate that both phages possess strong anti-*S. aureus* activity, although host-range differences exist against clinical isolates.

**Figure 3:**
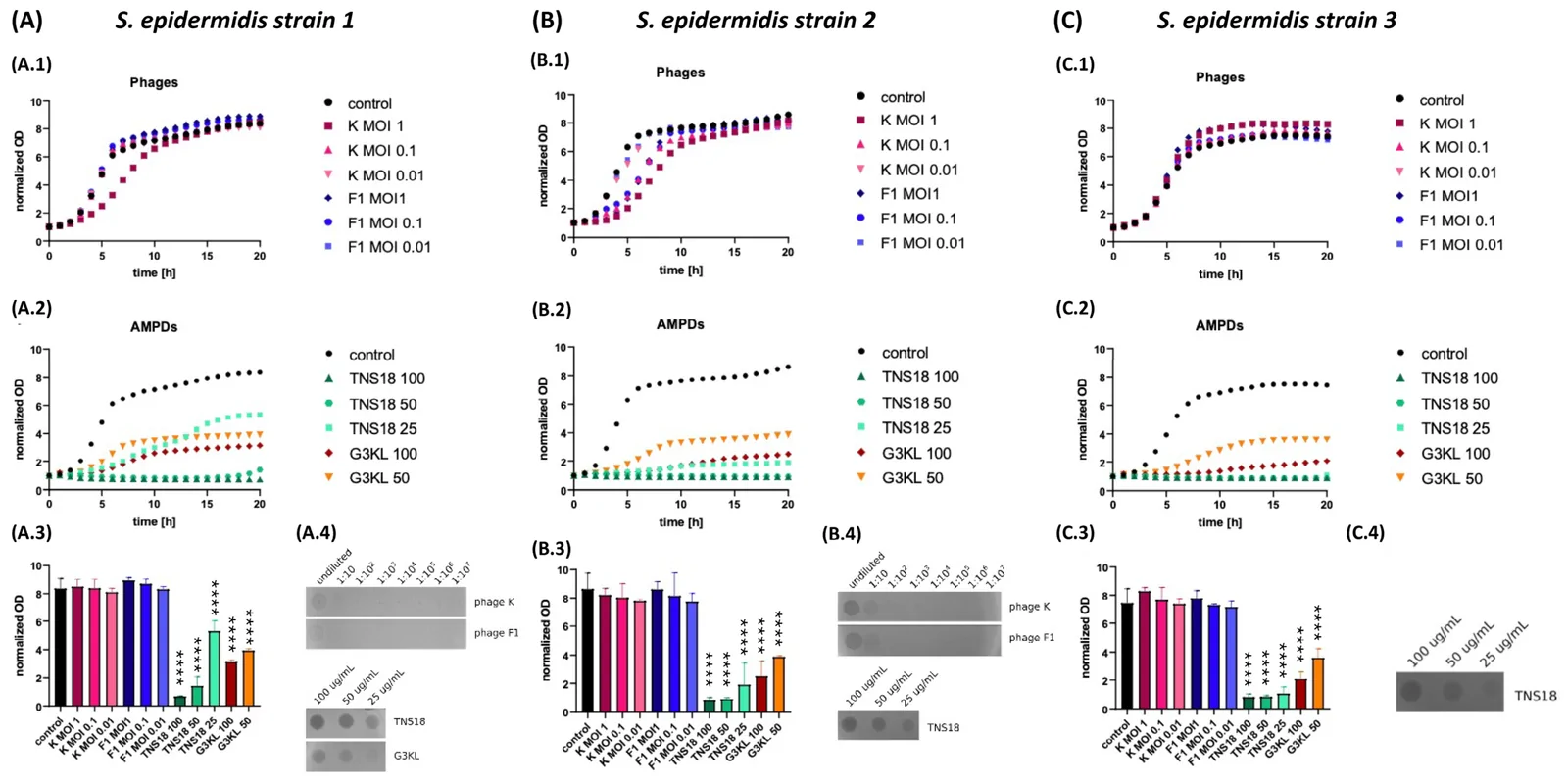
Susceptibility of *S. epidermidis* clinical isolates to bacteriophages and AMPDs. (**A.1**–**C.1**) Growth kinetics of *S. epidermidis* isolates incubated with Phage K or Phage F1 at multiplicities of infection (MOIs) of 1, 0.1, and 0.01, or without phage (control), over 20 h. OD_600_ was normalized to the initial value (t = 0 h). Data represent the mean of three biological replicates. (**A.2**–**C.2**) Growth kinetics of *S. epidermidis* isolates incubated with TNS18 (100, 50, and 25 μg/mL) or G3KL (100 and 50 μg/mL), or without AMPD (control), over 20 h. OD_600_ values were normalized to the initial value. Data represent the mean of three biological replicates. (**A.3**–**C.3**) Quantification of bacterial growth at 20 h for the conditions shown in panels (**A.1**–**C.1**). **** *p* ≤ 0.0001. (**A.4**–**C.4**) Drop test assay results showing the susceptibility of *S. epidermidis* isolates to serial dilutions of Phage K and Phage F1 and to TNS18 (100, 50, and 25 μg/mL) and G3KL (100 and 50 μg/mL).

In contrast, neither Phage K nor Phage F1 showed detectable activity against *S. epidermidis* isolates (Figure 3). No lysis zones were observed in drop tests, and bacterial growth kinetics remained comparable to untreated controls across all MOIs tested.

The AMPDs displayed a complementary activity profile. Against *S. aureus*, neither TNS18 nor G3KL generated visible lysis zones in drop tests, and only modest growth inhibition was observed in liquid culture (Figure 2). Conversely, *S. epidermidis* isolates were highly susceptible to dendrimer treatment, particularly to TNS18 (Figure 3). At a concentration of 100 μg/mL, TNS18 reduced bacterial growth by approximately one order of magnitude and produced clear zones of inhibition in drop tests. G3KL exhibited weaker and more variable antibacterial activity across the tested isolates. 

### 3.3. Development and Characterization of an In Vitro Staphylococcal Biofilm Model for Evaluating Implant-Associated Antimicrobial Strategies

To establish an in vitro model suitable for assessing antimicrobial candidates intended for implant-associated applications, biofilm formation by clinical *S. aureus* and *S. epidermidis* isolates recovered from PJIs was evaluated under a range of culture conditions. Parameters investigated included growth medium composition, glucose supplementation, NaCl concentration, and bacterial inoculum size.

**Figure 4:**
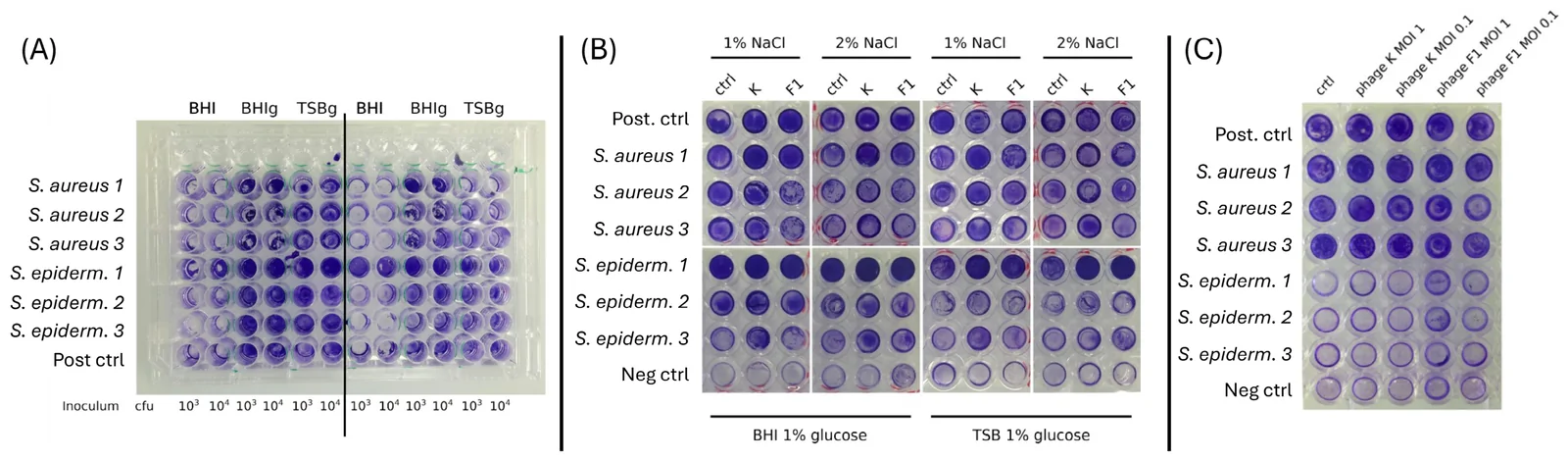
Optimization of a staphylococcal biofilm model and assessment of phage susceptibility. (**A**) Biofilm formation as shown by crystal violet staining for clinical *S. aureus* and *S. epidermidis* isolates under varying culture conditions, including different growth media, glucose concentrations, and bacterial inoculum densities. (**B**) Effect of growth medium composition and NaCl supplementation on biofilm formation and susceptibility to bacteriophages K and F1. (**C**) Biofilm formation and phage susceptibility of clinical isolates cultured in DMEM, a mammalian cell culture medium relevant to implant-associated applications (BHIg: BHI supplemented with glucose; TSBg: TSB supplemented with glucose).

As shown in Figure 4A, biofilm formation increased in glucose-supplemented BHI (BHIg) compared with standard BHI. Greater biofilm formation was also observed when fresh medium was added after 24 h of incubation. In contrast, the initial bacterial inoculum had little effect on the amount of biofilm detected. Biofilm formation was observed for all tested isolates. However, biofilms produced by all three *S. aureus* isolates were extensively disrupted during the washing steps of the crystal violet staining procedure. The three *S. epidermidis* isolates initially produced more stable biofilms that remained attached throughout the washing procedure, although subsequent experiments showed a partial disruption of biofilms formed by *S. epidermidis* isolates 1 and 2.

Biofilm formation was subsequently assessed in BHIg and TSBg supplemented with 1% or 2% NaCl. After 24 h of growth, biofilms were exposed to phages K or F1 at an MOI of 1 for 48 h. As shown by the untreated control wells in Figure 4B, substantial biofilm disruption occurred during the staining procedure under all tested conditions. No obvious differences in biofilm retention were observed between cultures supplemented with 1% or 2% NaCl.

Biofilm formation was also evaluated in DMEM supplemented with 10% FBS and 5% glutamine. After 48 h of incubation, biofilms were exposed to Phage K or Phage F1 at MOIs of 1 or 0.1 for 5 h. As shown in Figure 4C, all three *S. aureus* isolates formed detectable biofilms under these conditions, whereas none of the *S. epidermidis* isolates showed visible biofilm formation. Partial disruption of biofilms was again observed in the PBS-treated control wells following the staining procedure, making interpretation of phage-mediated anti-biofilm activity difficult to evaluate.

## 4. Discussion

The prevention of PJIs remains a major challenge in orthopedic surgery, particularly in the context of increasing AMR and the widespread ability of staphylococci to form biofilms on implant surfaces. In the present study, we evaluated two bacteriophages and two AMPDs as potential candidates for future implant-associated antimicrobial delivery systems. Our findings demonstrated that these antimicrobial classes exhibit complementary activity profiles against the two major staphylococcal species implicated in PJIs, namely *S. aureus* and *S. epidermidis*.

Bacteriophages K and F1 showed strong antibacterial activity against the tested *S. aureus* isolates, although strain-dependent differences in susceptibility were observed. While Phage K effectively lysed two of the three isolates, Phage F1 displayed activity against all strains. These findings are consistent with the inherent host specificity of bacteriophages and illustrate how relatively small differences between phages can influence the host range [11,12,13,14,15,16]. Interestingly, Phage K has previously been reported to infect both coagulase-positive and coagulase-negative staphylococci, yet no activity was observed against the small number of *S. epidermidis* isolates included in this study [20,30]. Although the mechanisms underlying this inefficacy remain unclear, differences in cell wall composition and glycan architecture between *S. aureus* and *S. epidermidis* likely influence phage adsorption and infection efficiency. N-acetylglucosamine has been reported to be involved in Phage K adsorption [31], yet this molecule is present in both *S. aureus* and *S. epidermidis* cell walls [32,33], suggesting that additional structural determinants may contribute to susceptibility. Indeed, phage approaches against coagulase-negative staphylococci are more difficult due to greater strain diversity [34].

The investigated AMPDs displayed the opposite species susceptibility pattern. Whereas *S. aureus* isolates exhibited only limited growth inhibition in the presence of AMPDs G3KL and TNS18, *S. epidermidis* isolates were highly susceptible, particularly to TNS18. A possible explanation for this observation lies in differences in bacterial cell surface properties. Specifically, AMPDs are positively charged molecules that interact with negatively charged bacterial membranes [17,18,19,35]. Previous studies have shown that *S. epidermidis* generally possesses a more negatively charged cell surface and lower membrane hydrophobicity than *S. aureus*, increasing its susceptibility to cationic antimicrobial agents [36]. Although the precise mechanism was not investigated in the present study, these differences may contribute to the observed contrasting susceptibility profiles.

Because biofilm formation is central to the pathogenesis of PJIs, establishing a robust in vitro biofilm model represented one of the objectives of this work. Glucose supplementation increased biofilm formation across several experimental conditions, consistent with the known contribution of extracellular polysaccharides, particularly polysaccharide intercellular adhesin (PIA), to staphylococcal biofilm development [6,9]. However, substantial variability in biofilm stability was observed among strains, and extensive biofilm disruption occurred during the crystal violet staining procedure. The limited number of strains available for this study may also have limited the biofilm readout, and further identification of strong biofilm formers could decrease the impact of other parameters of the assay. These limitations prevented the reliable quantification of antimicrobial effects against established biofilms. We chose to include this experience herein to highlight the difficulty in reliably and reproducibly measuring antibiofilm activity.

The results also highlighted the complexity of reproducing clinically relevant implant-associated biofilms in vitro. Although increasing NaCl concentrations from 1% to 2% did not improve biofilm stability, previous studies have reported enhanced biofilm formation at substantially higher salt concentrations, particularly in *S. aureus* and *S. epidermidis* [37,38]. Similarly, biofilm formation in vivo occurs in the presence of host proteins and coagulation factors that are largely absent from conventional laboratory media. The observation that *S. aureus*, but not *S. epidermidis*, formed biofilms in DMEM supplemented with FBS suggests that species-specific mechanisms of biofilm formation may influence performance under physiologically relevant conditions. Coagulase-mediated conversion of fibrinogen to fibrin has been shown to contribute to *S. aureus* biofilm formation on biomaterial surfaces and may represent a factor influencing the differences observed in this study [39,40,41].

Recent studies have highlighted the challenges of developing clinically relevant biofilm models for orthopedic applications [42,43,44]. Conventional static assays often fail to replicate the complex implant microenvironment, where bacterial communities interact with host cells, proteins, and biomaterial surfaces. Moreover, biofilm formation and antimicrobial susceptibility are strongly influenced by the underlying material and culture conditions. Together, these factors contribute to the variability observed across studies and underscore the need for more robust and physiologically relevant models to evaluate novel antimicrobial strategies for implant-associated infections.

Finally, bacterial adhesion and biofilm development are strongly influenced by surface chemistry, roughness, hydrophilicity, and protein adsorption, all of which vary substantially between biomaterials [6,8]. In this study, tissue-culture polystyrene plates were used as an initial standardized platform to screen for biofilm formation and antimicrobial susceptibility under controlled conditions. However, this model does not fully reproduce the physicochemical properties of orthopedic implant materials. Future studies should validate these findings on clinically relevant biomaterials, including titanium or cobalt-chromium alloys, before translation toward implant-associated antimicrobial coatings. Future studies should also consider more advanced co-culture and three-dimensional biofilm models that incorporate osteoblasts, immune cells, and clinically relevant metallic implant surfaces, as these systems are increasingly recognized as more representative of the biological conditions encountered during implant-associated infections.

## 5. Conclusions

This study evaluated two bacteriophages (Phage K and Phage F1) and two AMPDs (G3KL and TNS18) as potential antimicrobial candidates for preventing PJIs. The results demonstrated complementary antibacterial activity spectra against the more frequent causative pathogens *S. aureus* and *S. epidermidis*. Most antimicrobials exhibited favorable cytocompatibility with FE002-Bone human progenitor osteoblasts, supporting their suitability for applications involving orthopedic biomaterials or cell-based therapies. While additional work is needed to determine anti-biofilm activity and the activity of the selected phages and AMPDs against broader epidemiological panels, the present findings provide a foundation for the development of combined antimicrobial strategies targeting multiple PJI pathogens, which would enable empiric, preventive use. The complementary activities observed between phages and antimicrobial peptide dendrimers suggest that their integration into multifunctional implant coatings or localized delivery systems may offer a promising approach for reducing implant-associated infections while maintaining compatibility with regenerative therapies, and therefore potentially better surface integration attributes of implanted materials within the human tissues. Future studies should focus on evaluating phage–dendrimer combinations against mature biofilms formed on clinically relevant implant materials and on developing biomaterial-based delivery platforms capable of sustained local antimicrobial release.

## Data Availability

Data of the study are contained within the article file.

## Abbreviations

The following abbreviations are used in this manuscript:

AMPD	Antimicrobial Peptide Dendrimer
AMR	Antimicrobial Resistance
BHI	Brain Heart Infusion
BHIg	Glucose-Supplemented Brain Heart Infusion
CABMM	Competence Center for Applied Biotechnology and Molecular Medicine
CFU	Colony-Forming Unit
CHUV	Centre Hospitalier Universitaire Vaudois (Lausanne University Hospital)
DAIR	Debridement, Antibiotics, and Implant Retention
DMEM	Dulbecco’s Modified Eagle Medium
FBS	Fetal Bovine Serum
MDR	Multidrug-Resistant
MOI	Multiplicity of Infection
NTA	Non-Traditional Antimicrobial
OD	Optical Density
ON	Overnight
PBS	Phosphate-Buffered Saline
PFU	Plaque-Forming Unit
PIA	Polysaccharide Intercellular Adhesin
PJI	Prosthetic Joint Infection
RP-HPLC	Reverse-Phase High-Performance Liquid Chromatography
TSB	Tryptic Soy Broth
TSBg	Glucose-Supplemented Tryptic Soy Broth
WHO	World Health Organization
